# Homoharringtonine Synergized with Gilteritinib Results in the Downregulation of Myeloid Cell Leukemia-1 by Upregulating UBE2L6 in FLT3-ITD-Mutant Acute Myeloid (Leukemia) Cell Lines

**DOI:** 10.1155/2021/3766428

**Published:** 2021-09-21

**Authors:** Jiayi Cai, Honghui Huang, Xiaoli Hu, Wenjing Lang, Wanbin Fu, Lan Xu, Zilong Qiu, Hua Zhong, Fangyuan Chen

**Affiliations:** ^1^Department of Central Lab, Renji Hospital, Shanghai Jiao Tong University School of Medicine, Shanghai 200127, China; ^2^Department of Hematology, Renji Hospital, Shanghai Jiao Tong University School of Medicine, Shanghai 200127, China; ^3^Shanghai General Hospital, Shanghai Jiao Tong University, Shanghai 200080, China; ^4^Center for Excellence in Brain Science and Intelligence Technology, Institute of Neuroscience, State Key Laboratory of Neuroscience, Chinese Academy of Sciences, Shanghai 200031, China

## Abstract

FMS-like tyrosine kinase 3 (FLT3) mutant acute myeloid leukemia (AML) occurs in approximately 30% of all AML patients and still has a poor prognosis. This study is directed to investigate gilteritinib in combination with homoharringtonine (HHT) on FLT3-ITD-mutant AML cell lines. In our study, we found that cell proliferation was dramatically suppressed by the combination of gilteritinib and HHT. This combination therapy decreased the mitochondrial membrane potential, finally inducing apoptosis. We demonstrated that gilteritinib downregulated the expression of FLT3 and downstream signaling, further decreased the mRNA level of myeloid cell leukemia-1 (Mcl-1). HHT and combination therapy could upregulate UBE2L6, which induced the degradation of Mcl-1 via ubiquitin-proteasome system. Knockdown of UBE2L6 could protect Mcl-1 from deprivation through the ubiquitin-proteasome system. These findings may provide a novel theoretical basis for the treatment of AML patients with FLT3-ITD mutations.

## 1. Introduction

Acute myeloid leukemia (AML) is a type of hematological malignancy that is characterized by the accumulation of immature cells in the bone marrow. FLT3 mutation, which is a very common mutation in AML, was seen in one-third of the newly diagnosed patients [1]. FLT3 internal tandem duplication (FLT3-ITD) and tyrosine kinase domain (TKD) mutations cause the constitutive activation of FLT3 and its downstream signaling pathways, such as PI3K/AKT/mTOR, RAS/MAPK, and STAT5. AML patients with FLT3-ITD mutations were reported to show a higher relapse rate and inferior overall survival (OS) relative to patients with FLT3-wt [2, 3]. Therefore, several efforts have been made in order to find out the targets of FLT3 mutated protein. The US FDA permitted the second-generation of FLT3 inhibitor, that is, gilteritinib, to be used for relapsed FLT3-AML patients in November 2018. Gilteritinib is a dural inhibitor of FLT3/AXL. It has been observed that gilteritinib shows medical action against TKD but is unsuccessful in the inhibition of KIT [4]. Monotherapy with FLT3 inhibitors is limited due to the development of resistance leading to leukemia relapse despite the high initial response rates [5]. Combination therapy can be used to acquire a later response.

Myeloid cell leukeimia-1 (Mcl-1) is an antiapoptotic member of Bel-2 family and it is extremely necessary in the maintenance of cell survival, and, it reacts with Bax and Bak, in order to stop apoptosis. Mcl-1 has a high expression rate because of the differentiation and survival signals, including JAK/STAT, PI3K/AKT, and MAPK-dependent pathways [6]. So far, patients who have FLT3-ITD genetic alterations were found to harbor greater expression levels of Mcl-1 [7]. HHT, homoharringtonine, is obtained from herbs. In the past 30–40 years, HHT-contained chemotherapies are being used in China on a large scale [8, 9]. HHT was reported to interrupt protein synthesis, which resulted in the inhibition of the growth of leukemia cells and the induction of apoptosis. Mechanistically, HHT stops the binding of substrate with 60S ribosomal subunit in the first set of translation [10]. It can be seen that semisynthetic HHT can start apoptosis by stopping synthesis of protein and initiating the fast downregulation of Mcl-1 in myeloid leukemia cells. Moreover, the combination treatment of sorafenib and HHT demonstrated clinical efficacy in FLT3-ITD-mutant AML [10, 11].

Even today, there is no discussion or study if AML could be cured by the combined use of HHT and gilteritinib. In the present study, we hypothesized that combining gilteritinib with HHT would have synergistic antileukemic activity in FLT3-ITD-mutant AML. So, on the basis of this study, we summarize that p-FLT3 degradation by gilteritinib caused the decrease in mRNA expression of Mcl-1 producing less MAPK/ERK and JAK/STAT pathways. Along with that, HHT administration might lessen the protein quantity of Mcl-1 through the proteasome-ubiquitin system by upregulating UBE2L6.

## 2. Materials and Methods

### 2.1. Cell Line and Reagents

MV4-11 cells were purchased from the Stem Cell Bank, Chinese Academy of Sciences (Shanghai, China). During the whole experiments, MV4-11 cells were not used if they were passed over 30 times. Molm13 cells were kindly provided by the Shanghai Jiao Tong University School of Medicine. The cells were then cultured individually at 37°C in Iscove's modified Dulbecco's medium (IMDM) and Roswell Park Memorial Institute 1640 (RPMI-1640). Gilteritinib was purchased from MedChemExpress and prepared as a 10 *μ*m stock solution and HHT bought from Mingshen Company (Hangzhou, China), and then it was prepared as a 2 *μ*m stock solution, which was stored at −20°C. Just before the experiment, both the solutions were watered down on the basis of concentration, which was required along with the culture media. Finally, MG-132 was bought from Sigma-Aldrich.

### 2.2. Cell Proliferation Assay

Cell Counting Kit-8 (CCK-8) (Dojindo, Kumamoto, Japan) was used to determine the cytotoxic effects of HHT and gilteritinib on AML cells. Basically, 2 × 10^4^ cells/well were deposited in a plate containing 96 wells along with 100 *μ*L of growth medium. Then, the cells were treated with either gilteritinib or HHT and a combination of gilteritinib and HHT for 48 hours in the specified amount of doses. After that, CCK-8 reagents (10 *μ*L/well) were added and incubated for three more hours, and the absorbance was calculated to be 450 nm. After that, results were calculated by three experiments and it was stated in the form of mean of living cells' percentage as compared to the untreated group. CompuSyn was used to calculate the relationship between gilteritinib and HHT by the use of the combination index (CI). If the value was less than 1.0, it means that there was synergism and a value more or equal to 1.0 means antagonistic effect.

### 2.3. Flow Cytometric Assay for Determining Apoptosis, Cell Cycle, and Mitochondrial Membrane Potential (MMP)

For examining apoptosis, cells were treated either with the combination of HHT and gilteritinib or singularly agent for 24 or 48 hours. This step was followed by a staining step where annexin-V/PI was used for staining. The staining was done at room temperature for 15 minutes and then was kept in dark as prescribed by the manufacturer. Then, JC-1 Fluorescent Probe Kit was used to calculate the MMP loss. Finally, cells were examined using flow cytometry (LSRFortessa, BD Biosciences).

### 2.4. Western Blot Analysis

After the completion of treatment, around 2 × 10^6^/ml cells were disintegrated in one condition. After that, BCA Protein Assay Kit (Beyotime Company, Shanghai, China) was used to calculate the protein concentration. It is evaluated that whole cell lysates were then electrophoresed in 8–12 percent SDS-PAGE before being put into polyvinylidene difluoride membrane (Millipore, Burlington, MA, USA). The blots were then envisioned with the help of Odyssey CLx imaging system by ECL reagents (Yeasen Biotech Co., Ltd., Shanghai, China).

### 2.5. Microarray Analysis

Total RNA was quantified using the NanoDrop ND-2000 system (Thermo Fisher Scientific), and the RNA integrity was assessed using the Agilent Bioanalyzer 2100 (Agilent Technologies, Santa Clara, CA, USA). Sample labeling, microarray hybridization, and washing were performed based on the manufacturer's instruction. In brief, total RNA was transcribed to double-stranded complementary DNA, then synthesized into complementary RNA, and labeled with cyanine-3-CTP. The labeled complementary RNAs were then hybridized onto the microarray. After washing, the arrays were scanned with the Agilent G2505C scanner (Agilent Technologies). The Feature Extraction software (version 10.7.1.1; Agilent Technologies) was used to analyze array images to obtain raw data. Genespring (version 14.8; Agilent Technologies) was employed to finish the basic analysis with the raw data. To begin with, the raw data were normalized with the quantile algorithm. The probes suggesting that, at least one out of two conditions had flags in “detected” were chosen for further data analysis. Differentially expressed genes were then identified through fold-changes as well as p values calculated with the t-test. The threshold set for up- and downregulated genes was a fold change of at least 2.0 and a p value of 0.05 or less. Afterward, Gene Ontology analysis and Kyoto Encyclopedia of Genes and Genomes analysis were applied to determine the roles of these differentially expressed mRNAs. Finally, hierarchical clustering was performed to display the distinguishable genes' expression patterns among samples. The Agilent SurePrint G3 Human Gene Expression v3 8x60K Microarray (072363; Agilent Technologies) was used in this experiment, and data analysis of the 12 samples was conducted by OE Biotechnology Co., Ltd. (Shanghai, China)

### 2.6. Quantitative Real-Time Polymerase Chain Reaction (qRT-PCR) Analysis

The Qiagen RNeasy Mini Kit (Hilden, Germany) was used to extract RNA according to the manufacturer's instructions. Then, the TransScript FirstStrand CDNA Synthesis Super Mix Kit was used to reverse-transcribe the RNA. Then, Step One Plus real-time PCR system was used for qPCR research, and the purpose is to determine the level of gene expression. The gene expression level is normalized with GAPDH. The primer sequences of indicated genes used are listed in Table 1.

### 2.7. RNA Interference Experiments

A small interfering RNA (siRNA), which was UBE2L6-specific or a nonspecific siRNA control (NC siRNA), was chemically created by Ribobio Company (Guangzhou, China). First, 100 pmol of siRNA was introduced to 1 × 10^6^ of MV4-11 and Molm13 cells with the help of HidffTrans suspension cell-free liposomal transfection reagent (Yeasen Biotech Co., Ltd.) as per the manufacturer's instructions. The transfected cells were incubated for 24 hours, and then they were collected to undergo qRT-PCR analyses to measure the UBE2L6 expression level or for further experiments.

### 2.8. Statistical Analysis

It is analyzed that all the data taken for the program and research is considered as mean and standard error and also followed with the independent experiment and selecting the gathering of the data. The *t*-tests undertaken were used to assess the determined program process for the experimental results, which is further deployed with the outcomes. The unpaired *t*-tests were used to determine the statistical significance of experimental results between various conditions. Using the GraphPad Prism version 6 software program (La Jolla, CA, USA), statistical analysis was done, and the *p* value equal to or less than 0.05 was regarded as statistically important.

## 3. Results

### 3.1. Cotreatment with Gilteritinib and HHT Suppressed Cell Proliferation by Inducing Apoptosis in FLT3-ITD-Mutated Cell Lines

**Figure 1:**
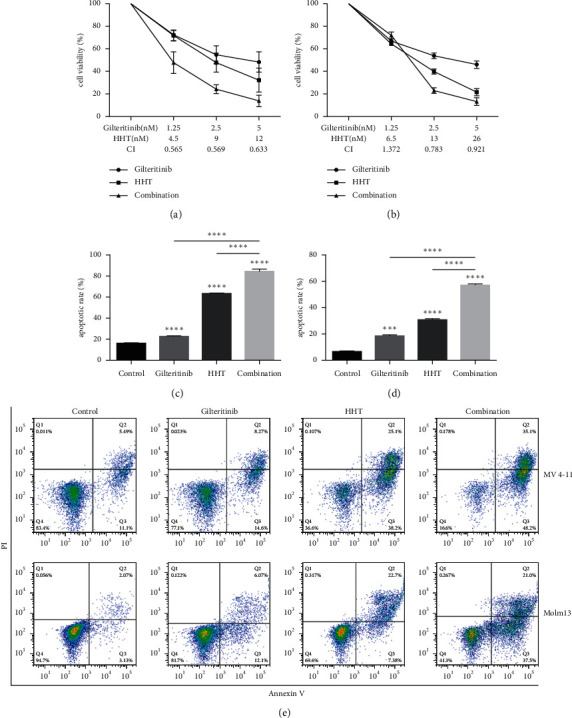
HHT synergized with gilteritinib to suppress cell viability and induced apoptosis in FLT3-ITD mutant cell lines. (a) Cell viability of MV4-11 cells after treating with gilteritinib and HHT for two days, with CI values mentioned below the figure. (b) Cell viability of Molm13 cells after treating with gilteritinib and HHT for 48 hours, with CI values listed under the figure. (c, d) Apoptosis rates of MV4-11 cells (c) and Molm13 cells (d) treated with gilteritinib and/or HHT for 48 hours. DMSO-treated cells served as a control. Then, costaining of cells was done with annexin-V and PI, and the measurement of apoptosis was done by flow cytometry (e). CI: combination index; CI < 1 indicated these two drugs had a synergetic effect.  ^*∗∗∗*^*p* < 0.005 and  ^*∗∗∗∗*^*p* < 0.001.

First, the measurement was done to determine whether the combination of gilteritinib and HHT would have an interactive effect on MV4-11 and Molm13 cells. To this end, MV4-11 and Molm13 cells were treated with indicated concentrations of gilteritinib and HHT, and cell viability was analyzed using the CCK-8 assay. For MV4-11 cells, after being stimulated with gilteritinib, the cell viability was 72.57 ± 1.73%, 54.80 ± 7.95%, and 48.35 ± 9.04%. The cell viability was 71.74 ± 4.71%, 47.82 ± 8.45%, and 32.28 ± 10.68% after the stimulation with HHT. After being treated with different concentrations of gilteritinib, the cell viability of Molm13 cells was 66.77±%, 53.77 ± 2.42%, and 45.87 ± 3.36%, separately. The cell viability of Molm13 cells after treatment with HHT was 64.33 ± 1.85%, 39.70 ± 2.16%, and 21.53 ± 2.05%. As shown in Figures 1(a) and 1(b), the combination of gilteritinib and HHT led to noticeable and improved growth inhibition in MV4-11 and Molm13 cells. CI values were then obtained using the CompuSyn software, indicating synergism between 2.5 nm of gilteritinib and 9 nm of HHT in MV4-11 cells and 2.5 nm of gilteritinib and 13 nm of HHT in Molm13 cells (Figures 1(a) and 1(b)). After the combination treatment, the cell viability of MV4-11 cells was 47.95 ± 9.50%, 24.36 ± 3.90%, and 13.88 ± 5.12%, and the cell viability of Molm13 cells was 71.93 ± 2.90%, 22.87 ± 2.58%, and 13.23 ± 3.37%. To ensure if cytotoxicity of HHT and gilteritinib was related to the start of apoptosis, the apoptotic cell percentage was calculated by annexin-V/PI double staining method. As shown in Figures 1(c)–1(e), every treatment with one agent leads to a moderate increase in apoptosis, while the percentage was higher when the combination of HHT and gilteritinib was used. After 48 hours, the apoptotic rates of combination treatment were 84.40 ± 2.17% and 58.33 ± 1.46% in MV4-11 and Molm13 cells, respectively. Altogether, these findings recommended that HHT synergized with gilteritinib to suppress cell viability and induce apoptosis in FLT3-ITD (+) cell lines.

### 3.2. Cotreatment with Gilteritinib and HHT Interrupted MMP and Activated the Intrinsic Apoptotic Pathway

**Figure 2:**
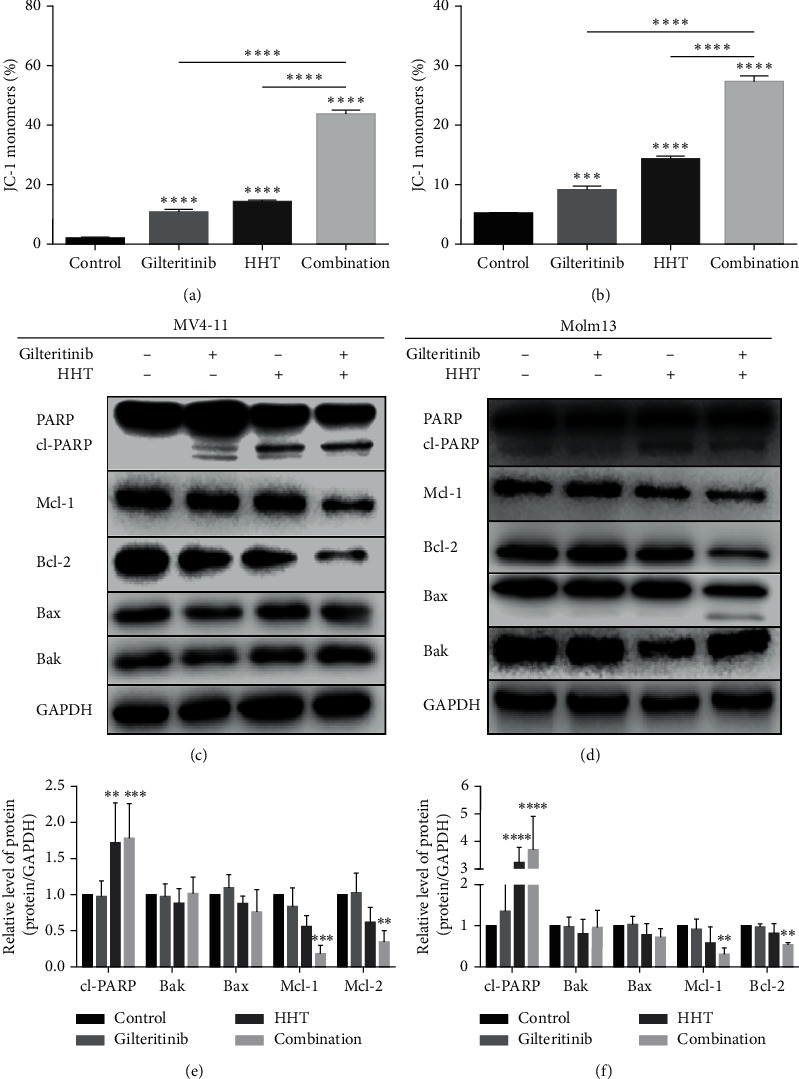
Cotreatment of gilteritinib and HHT impaired the MMP and activated an intrinsic apoptotic pathway. (a, b) Percentage of JC-1 monomers. MV4-11 cells (a) and Molm13 cells (b) were given a treatment of gilteritinib and/or HHT for 24 hours. The cells treated with DMSO were used as a control group. (c, d) Western blot analysis of PARP, cleaved PARP, Mcl-1, Bcl-2, Bax, and Bak in treated MV4-11 cells (c) and Molm13 cells (d). (e, f) Quantification of (c) and (d); each group was equated with the control group.  ^*∗*^*p* < 0.05,  ^*∗∗*^*p* < 0.01, and  ^*∗∗∗*^*p* < 0.005.

The apoptotic signaling pathways include receptor-mediated (extrinsic) and mitochondria-mediated (intrinsic) pathways. Next, MMP assay was conducted to find out if the apoptosis of MV4-11 and Molm13 cells, after the treatment with gilteritinib and HHT, occurred through a mitochondria-mediated pathway. The results revealed that gilteritinib or HHT alone could slightly cause the loss of MMP, while the combination of both resulted in significant loss of MMP after 24 hours (Figures 2(a) and 2(b)). Furthermore, the mechanism of the intrinsic apoptotic pathway was investigated by western blotting of apoptosis-related proteins. As visible in Figures 2(c)–2(f), the protein amount of cleaved PARP was significantly increased in MV4-11 and Molm13 cells after treatment with either only HHT or with the combination of HHT and gilteritinib. Also, the number of prosurvival proteins Mcl-1 and Bcl-2 was reduced significantly, especially in MV4-11 cells. However, there were no obvious changes in the number of proapoptotic proteins Bak and Bax in MV4-11 cells. Meanwhile, in Molm13 cells, the expression of Bax was moderately decreased without a significant difference following combination treatment with gilteritinib and HHT.

So far, these results indicated that the combination of gilteritinib and HHT therapy in leukemia cells could induce an intrinsic apoptosis pathway by disrupting the mitochondrial membrane potential.

### 3.3. The Combination of Gilteritinib and HHT Downregulated Mcl-1 Expression via Different Mechanisms

As shown earlier, gilteritinib or HHT alone could slightly reduce the manifestation of the antiapoptotic protein Mcl-1, while cotreatment could significantly downregulate the levels of protein of Mcl-1. It was reported that alteration or mutation in FLT3-ITD led to an abnormal change in its further kinases like STAT5, AKT, and MAPK/ERK [12]. Some of these pathways are occupied in the start of Mcl-1 transcription by affecting the particular transcription factor response elements that bind to Mcl-1 promoter.

**Figure 3:**
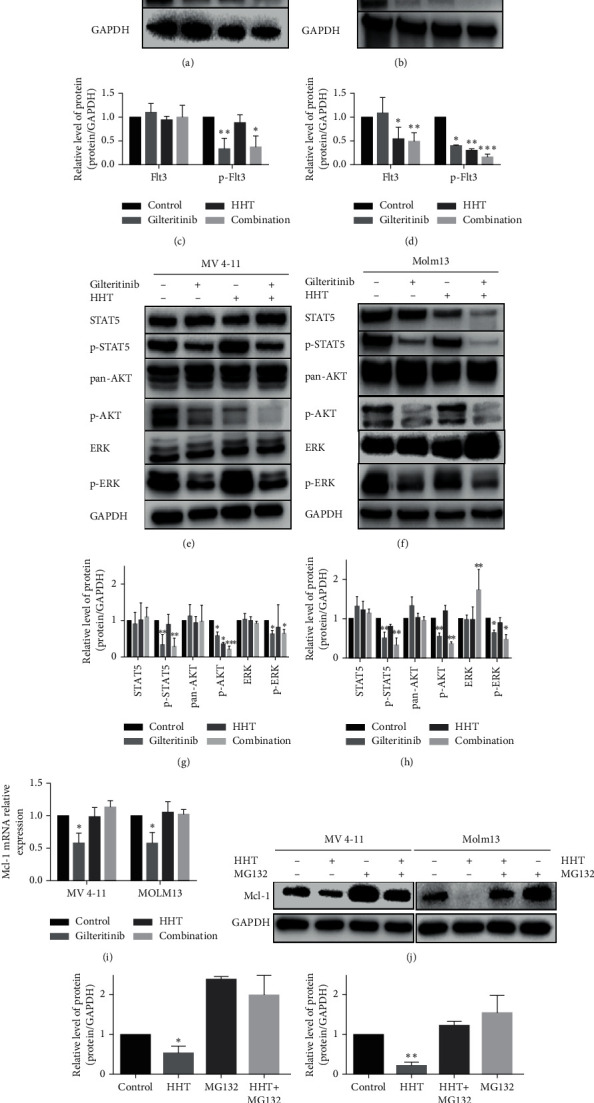
The combination of gilteritinib and HHT downregulated Mcl-1 expression via different mechanisms. (a, b)The protein level of FLT3 and p-FLT3 in MV4-11 cells and Molm13 cells was altered by gilteritinib and/or HHT. (c, d) Quantification of (a) and (b). The effect of gilteritinib and/or HHT on the downstream signaling of FLT3 in MV4-11 cells (e) and Molm13 cells (f). (g, h) Quantification of (e) and (f). (i) The mRNA expression of Mcl-1 was measured by qPCR in MV4-11 cells and Molm13 cells treated with HHT and/or gilteritinib at the indicated concentrations for 24 hours. (j) The expression levels of Mcl-1 and GAPDH were analyzed by western blotting in MV4-11 and Molm13 cells treated with HHT and/or MG-132 (10 μM) for four hours. (k, l) Quantification of (j). Each group was compared with the control group.  ^*∗*^*p* < 0.05,  ^*∗∗*^*p* < 0.01, and  ^*∗∗∗*^*p* < 0.005.

To explain the underlying process of the reduced expression of Mcl-1, whether the coadministration of gilteritinib and HHT could synergistically affect FLT3-ITD-mutant protein, aberrant downstream signaling was first explored by treating MV4-11 and Molm13 cells with 2.5 nm of gilteritinib or 9 nm (MV4-11 cells) or 13 nm (Molm13 cells) of HHT or by coadministration of these two agents for 48 hours and then performing western blotting. The results revealed that gilteritinib alone dramatically decreased phosphorylated protein levels of FLT3, STAT5, ERK, and AKT but did not disturb their total level of protein. However, the combination of gilteritinib and HHT did not have a synergistic influence on the reduction of FLT3-ITD-mutant protein and aberrant downstream signaling except for p-AKT in the MV4-11 cell line (Figures 3(a), 3(c), 3(e), and 3(g)). The effects of gilteritinib and/or HHT on Molm13 cells were slightly different from those on MV4-11 cells. HHT and cotreatment of HHT and gilteritinib resulted in reduced protein levels of p-FLT3 and p-STAT5 together with total protein levels of FLT3 and STAT5. Cotreatment of HHT and gilteritinib in Molm13 cells initiated a major growth in the total protein amount of ERK (Figures 3(b), 3(d), 3(f), and 3(h)). Next, qPCR was performed to elucidate whether gilteritinib or/and HHT could affect the mRNA expression of Mcl-1. The indication provided by the result was that mRNA expression of Mcl-1 declined after treating with gilteritinib but the alone and combined treatment of HHT did not affect the mRNA expression of Mcl-1 (Figure 3(i)). Altogether, these results propose that gilteritinib disrupts FLT3-ITD-mutant protein and aberrant downstream signaling, resulting in the reduced expression of Mcl-1 mRNA.

However, there was no effect of HHT on mRNA level of Mcl-1 but it did reduce its protein level. Hence, we postulated that HHT might degrade the Mcl-1's protein level at the posttranscriptional stage, such as via the ubiquitin-proteasome system. Next, MV4-11 and Molm13 were treated with MG-132, an inhibitor of proteasome, and then HHT was applied for four hours. Then, western blotting was done of Mcl-1 protein. As shown in Figures 3(j)–3(l), MG-132 might stop the deprivation of Mcl-1 after HHT treatment, and this suggested that HHT could mediate the deprivation of Mcl-1 by the pathway of ubiquitin-proteasome. Interestingly, there was an almost complete depletion in the protein amounts of Mcl-1 in Molm13 cells after HHT treatment for 4 h (Figure 3(k), right part).

### 3.4. The Combination of Gilteritinib and HHT Upregulated UBE2L6 Expression

**Figure 4:**
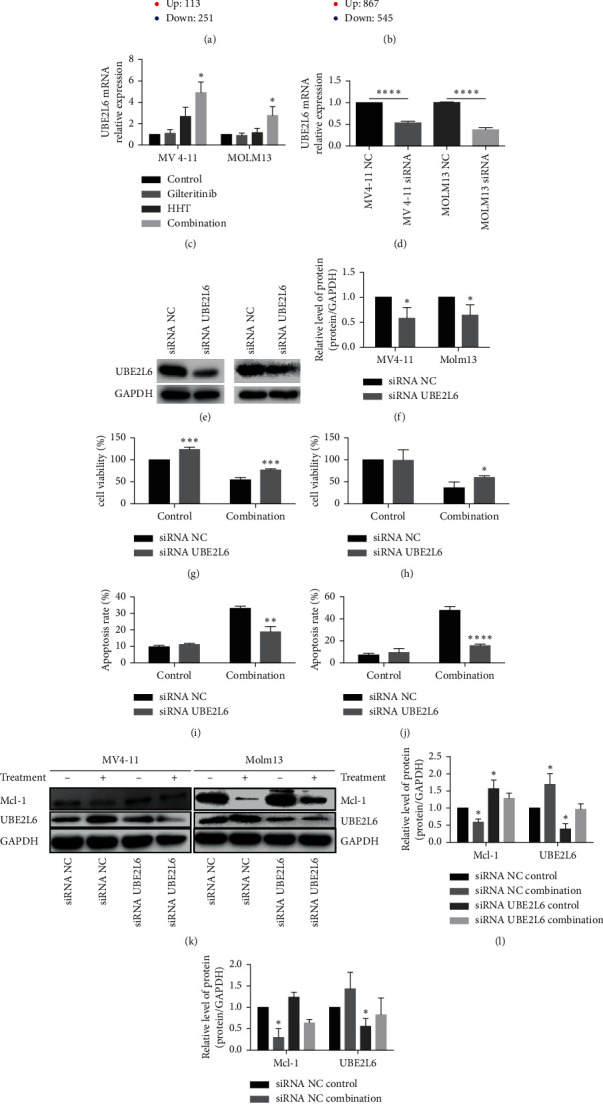
The combination of gilteritinib and HHT resulted in Mcl-1 degradation by upregulated UBE2L6 expression. (a, b) Volcano plot of differential mRNAs measured by microarray in MV4-11 cells treated with 9 nM of HHT and/or 2.5 nM of gilteritinib.(c) The mRNA expression of UBE2L6 was measured by qPCR in MV4-11 cells and Molm13 cells treated with HHT and/or gilteritinib at the indicated concentrations for 24 hours. (d) The mRNA expression of UBE2L6 was measured by qPCR in MV4-11 cells and Molm13 cells after siRNA transfection. (e) The protein level of UBE2L6 was measured by western blotting in MV4-11 cells (left panel) and Molm13 cells (right panel) after siRNA transfection. (f) Quantification of (e). (g, h) Cell viability of Molm13 (g) and MV4-11 cells (h) treated with gilteritinib and HHT after siRNA transfection. (i, j) Apoptosis rates of Molm13 (i) and MV4-11 cells (j) treated with gilteritinib and HHT after siRNA transfection. (k) Mcl-1, UBE2L6, and GAPDH protein levels of MV4-11 and Molm13 cells treated with gilteritinib and HHT after siRNA transfection. (l, m) Quantification of (k). Each group was compared with control group.  ^*∗*^*p* < 0.05,  ^*∗∗*^*p* < 0.01, and  ^*∗∗∗*^*p* < 0.005

To analyze possible target genes influenced by HHT therapy alone and combined therapy using HHT and gilteritinib, microarray analysis was used to compare mRNA expression before and after the indicated treatment in MV4-11 cells. In total, 113 different genes were increased (by more than twofold) and 251 different genes were decreased (by less than twofold) by HHT treatment. After combined treatment, 867 different genes were increased (by more than twofold) and 545 different genes were decreased (by less than twofold) (Figures 4(a) and 4(b)). UBE2L6, an E2-conjugase, was increased by about twofold by HHT and by 2.5-fold by combined treatment using HHT and gilteritinib. We performed qPCR to further confirm this finding. We authenticated that UBE2L6 was upregulated by four-to-sixfold by treatment using HHT or the combination of HHT and gilteritinib (Figure 4(c)). This result was also observed in Molm13 cells.

To confirm whether the increase in production of UBE2L6 led to the degradation of Mcl-1 in AML cells, after gilteritinib and HHT treatment, the mRNA expression was suppressed with the help of siRNA in MV4-11 and Molm13 cell lines (Figure 4(d)). The protein levels of UBE2L6 in MV4-11 and Molm13 cells after transfection with siRNA were then measured by western blotting (Figures 4(e) and 4(f)). MV4-11 and Molm13 cells transfected with siRNA counter to UBE2L6 or a siRNA control were later treated with gilteritinib and HHT for 48 hours. The effect of this combination treatment on Mcl-1 downregulation was diminished in UBE2L6-knockdown MV4-11 and Molm13 cells (Figures 4(k)–4(m)). Consequently, upon cotreatment with gilteritinib and HHT, there was an increase in cell viability after UBE2L6 knockdown (Figures 4(g) and 4(h)). To investigate the effects of UBE2L6 suppression on the MV4-11 and Molm13 cells' apoptosis, the apoptotic cells' percentage was examined by annexin-V/PI assay. The results indicated that the apoptotic cells were also reduced in UBE2L6 suppression cells with treatment of gilteritinib and HHT at the same concentrations (Figures 4(i) and 4(j)). These results and findings further convinced that HHT combined with gilteritinib upregulated UBE2L6, which increased the degradation of Mcl-1 and then initiating the apoptosis of FLT3-ITD mutant AML cells.

## 4. Discussion

In this study, the synergistic effect of gilteritinib and HHT and the underlying mechanisms in FLT3-ITD mutated AML cell lines were evaluated. We demonstrated the combination of gilteritinib and HHT as therapy exhibited important antileukemic effects in vitro. In particular, this combination therapy synergistically inhibited Mcl-1 through different mechanisms leading to apoptosis.

FLT3-ITD mutations start the constitutive activation of FLT3 and its downstream signaling pathways, such as PI3K/AKT/mTOR, RAS/MAPK, and STAT5 [12]. US FDA permitted the second generation FLT3 inhibitor, that is, gilteritinib, to be used for the treatment of relapsed FLT3-ITD(+) AML in adults, in November 2018. Gilteritinib is a dural inhibitor of FLT3/AXL, which also provides significant action against TKD gene alterations but fails to do so for KIT [4]. Although FLT3-TKI-based regimens have exhibited encouraging response in both the relapsed/refractory settings and frontline, several patients are still unsuccessful to react to FLT3 inhibitors or subsequently relapse [13]. For the betterment of these results, a better understanding of the underlying mechanism of the drug resistance should be done. More production of proteins, which are antiapoptotic such as Bcl-2, Bcl-xL, and Mcl-1, is usually seen in TKI-resistant cases, and this might contribute to a phenotype that is resistant [13]. Around 37% of patients who failed at least one prior FLT3 inhibitor achieved a response with gilteritinib, suggesting the potential for this drug to overcome resistance to other TKIs, suggesting that it could still be effective in patients who have relapsed or who are refractory to induction with midostaurin [14]. Mori et al. demonstrated that gilteritinib blocked FLT3 phosphorylation and further impaired the phosphorylation of ERK, STAT5, and AKT, and all of them are downstream targets of FLT3 activation. Consequently, gilteritinib could inhibit AML cell proliferation in both in vivo and in vitro models [4]. In agreement with the work of Masamichi and colleagues, we found that gilteritinib alone can reduce the phosphorylation of FLT3 and suppress the phosphorylation of downstream targets without impairing the total level of FLT3. The Inhibition setup of the aberrant downstream signaling might finally end in the reduction of the mRNA expression of Mcl-1.

Recently, Jin's laboratory revealed that HHT had a more sensitive cytotoxic effect on FLT3 mutant AML cells. [15]. Therefore, we guessed if any antileukemic effect could be produced when HHT and gilteritinib are combined. As anticipated, gilteritinib and HHT synergistically inhibited the growth of FLT3 mutant AML cell lines and induced apoptosis. This combination therapy could impair MMP and activate an intrinsic apoptotic pathway. Furthermore, we found that gilteritinib and HHT could downregulate Mcl-1 via different pathways. As shown in Figure 4, gilteritinib can block the aberrant downstream signaling of FLT3, which led to the downregulation of Mcl-1 in terms of mRNA level. HHT alone did not affect FLT3 and its downstream signaling, but the inhibition of Mcl-1 was increased by combining gilteritinib with HHT. HHT could upregulate the mRNA level of UBE2L6, which might induce the ubiquitin of Mcl-1, finally promoting the deprivation of Mcl-1 through the ubiquitin-proteasome system and inducing apoptosis in vitro. The upregulation of UBE2L6 was enhanced by combination therapy using gilteritinib and HHT, and this may be the mechanism behind how the combination of gilteritinib and HHT exhibited a substantial antileukemic action on FLT3-ITD mutant AML cell lines. Interestingly, there was an almost complete depletion in the protein level of Mcl-1 in Molm13 cells after treated with HHT for 4 h (Figure 3(k) right part). The mechanism of quick action of HHT may be the inhibition of protein synthesis and the consequent depletion of short-lived proteins such as Mcl-1.

Ma et al. presented that the amalgamation of gilteritinib/midostaurin and venetoclax exhibited notable antileukemic synergy in FLT3-ITD AML cells and primary samples of patients by less production of Mcl-1. FLT3 inhibitor induced the downregulation of Mcl-1, improving venetoclax activity. The phosphorylated expression of ERK is encouraged by venetoclax while stopped if it is paired with midostaurin or gilteritinib. Concurrent less production of Mcl-1 by midostaurin or gilteritinib and inhibition of Bcl-2 by venetoclax ends in “free” Bim, causing the synergistic induction of apoptosis. Gilteritinib coupled with venetoclax offers therapeutic promise, according to in vivo data [14]. It was pointed out that the mechanism of venetoclax resistance is partly attributable to Mcl-1 overexpression [16]. We discovered that HHT may downregulate both Bcl-2 and Mcl-1 in our investigation, confirming HHT's powerful anti-AML effects. The efficacy and safety of venetoclax for elderly AML patients are quite good [17, 18]. So, we suspected that HHT might be used as an alternative choice for venetoclax.

## 5. Conclusions

In conclusion, our results demonstrated that gilteritinib alone could significantly inhibit aberrant downstream signaling, including the JAK/STAT and MAPK/ERK pathways. Combining gilteritinib with HHT could induce an intrinsic apoptotic pathway, further inhibiting the cell proliferation in FLT3-ITD mutated cell lines. Gilteritinib inhibited the the mRNA level of Mcl-1; however, HHT and combined therapy promoted the deprivation of Mcl-1 through the ubiquitin-proteasome system by upregulating UBE2L6. Combining gilteritinib and HHT had a synergized effect on FLT3-ITD-mutated AML cell lines. These findings support us to further investigate the synergetic effect of gilteritinib and HHT on animal models. It needs to be further estimated whether the combination of these two drugs might have a synergetic effect on AML patients with FLT3-ITD mutations, so we are going to carry out a clinical trial on gilteritinib and HHT for R/R AML patients with FLT3-ITD mutation.

## Figures and Tables

**Table 1:** The primer sequences of indicated genes.

Gene name	Primer sequence
Mcl-1	F: 5′-CACAGTGACGCTTCCTGAAAC-3′
	R: 5′-GCCATCATTAGGATCTGGGAGA-3

UBE2L6	F: 5′- AGCTGGAGGATCTTCAGAAGA-3′
	R: 5′- TGGTTGTGAATTTGATCATGGG-3

GAPDH	F: 5′-GGAGCGAGATCCCTCCAAAAT-3′
	R: 5′-GGCTGTTGTCATACTTCTCATGG-3

## Data Availability

The data used to support the findings of this study are available from the corresponding author upon request.
